# Role of β-Interferon Inducer (DEAE-Dextran) in Tumorigenesis by VEGF and NOTCH1 Inhibition along with Apoptosis Induction

**DOI:** 10.3389/fphar.2017.00930

**Published:** 2017-12-19

**Authors:** Anita K. Bakrania, Bhavesh C. Variya, Snehal S. Patel

**Affiliations:** ^1^Department of Pharmacology, Institute of Pharmacy, Nirma University, Ahmedabad, India; ^2^Zydus Research Centre, Ahmedabad, India

**Keywords:** DEAE-Dextran, β-interferon, TNBC, anti-proliferative, apoptosis, angiogenesis, VEGF, NOTCH1

## Abstract

As a novel target for breast cancer, interferon inducers have found its role as anti-angiogenic agents with diethylaminoethyl dextran (DEAE-Dextran) being a molecule used for centuries as a transfection agent. Our results herein offer an explanation for the emergence of DEAE-Dextran as an anti-tumor agent for TNBC with in-depth mechanistic approach as an anti-angiogenic molecule. DEAE-Dextran has found to possess cytotoxic activity demonstrated during the various *in vitro* cytotoxicity assays; moreover, as an anti-oxidant, DEAE-Dextran has shown to possess excellent reactive oxygen species scavenging activity. The interferon inducing capacity of DEAE-Dextran was determined qualitatively as well as quantitatively specifically demonstrating overexpression of β-interferon. As a measure of anti-proliferative activity, DEAE-Dextran exhibited reduced ki67, p53, and PCNA levels. Also, overexpression of CK5/6 and p63 in DEAE-Dextran treated animals indicated improvement in breast cell morphology along with an improvement in cell–cell adhesion by virtue of upregulation of β-catenin and E-cadherin. Anti-angiogenic property of DEAE-Dextran was concluded by the downregulation of CD31, VEGF, and NOTCH1 both *in vivo* and *in vitro*. Further, apoptosis due to DEAE-Dextran, initially determined by downregulation of Bcl2, was confirmed with flow cytometry. Overall, results are defensive of DEAE-Dextran as an emerging anti-tumor agent with mechanisms pertaining to β-interferon induction with probable VEGF and NOTCH1 inhibition as well as apoptosis which still needs to be studied in further depth.

## Introduction

Breast cancer is a leading cause of cancer-related mortality in females globally. This malignancy represents a heterogeneous group of tumors with characteristic molecular features and diverse response to therapy (Banerji et al., 2012; Bakrania et al., 2016). Advances in breast cancer therapy, targets angiogenesis as a novel means of therapeutic approach for the disease especially in triple negative breast cancer where no specific targeted therapy is available till date (Bakrania et al., 2016). Angiogenesis has a major role in tumor growth, progression, and metastasis. This is as a circumstance of high demands of oxygen within the core, creating hypoxic conditions that further lead to the production of angiogenic factors for proliferation forming tubular vessels and sprouting, leading to a deeper circulatory network in the tumor (Kong et al., 2014; Bakrania et al., 2018).

As a new target for breast cancer, interferon inducers have found its role as anti-angiogenic agents. Till date, β-interferon has been approved for multiple sclerosis for the past two decades; however, β-interferon has also been recently studied in carcinoma cell lines and found to possess protective effect against tumor cells greater than α-interferon (Westcott et al., 2015).

Diethylaminoethyl dextran, a polycationic derivative of dextran offers a wide range of chemical and biological properties such as; adjuvant in vaccines, agent for transfection, agent for gene therapy, stabilizer for proteins, flocculent, and agent for drug delivery. DEAE-Dextran has also been reported to enhance production of interferons induced by polyinosinic–polycytidylic acid by various mechanisms; (1) it exerts a substantial protection of the inducer against extracellular RNase and, (2) it exerts a cell-mediated effect related to increased permeability for larger inducer molecules (Dianzani et al., 1969, 1971; Pitha and Carter, 1971). Hence, we have carried out various *in vitro* and *in vivo* studies in order to determine the interferon inducing capacity of DEAE-Dextran, as well as the in-depth mechanistic approach of DEAE-Dextran as a novel and emerging anti-cancer agent for TNBC.

## Materials and Methods

### Materials

Molecular biology grade reagents were commercially purchased. Thiazolyl Blue Tetrazolium Blue (MTT), trypan blue dye, H_2_DCFDA, 4′,6-Diamidino-2-phenylindole dihydrochloride (DAPI), DEAE-Dextran, 7,12-dimethyIbenz[a]anthracene (DMBA), and BCA protein estimation kit were purchased from Sigma–Aldrich (St. Louis, MO, United States). Dulbecco’s Modified Eagle’s Medium (DMEM), Leibovitz L-15 medium (L-15), Dulbecco’s Phosphate buffer saline (DPBS), and fetal bovine serum (FBS) were purchased from Invitrogen (Life Technologies, United States). Estrogen (ab32063), Progesterone (ab2765), HER2 (ab106575), CD31 (ab28364), ki67 (ab15580), p53 (ab131442), p63 (ab124762), CK5/6 (MA5-12429), bcl2 (ab59348), PCNA (ab18197), b-catenin (ab32572), and E-cadherin (ab133597) antibodies were purchased from Abcam, United Kingdom. β-Interferon (Relibeta) was purchased from Reliance, Pvt. Ltd., β-interferon ELISA kit was purchased from YH Biosearch Laboratory (China), Annexin V and propidium iodide were purchased from Thermo Fisher Scientific (United States). All other chemicals used were of analytical grade and purchased from Merck (Darmstadt, Germany).

#### Cell Lines and Culture

HEK293, MCF7, and MDA-MB-231 cell lines were generously provided by Zydus Research Centre, India (obtained from ATCC, United States). HEK293 and MCF7 cells were grown in DMEM culture media containing L-glutamine (2 mmol/l). MDA-MB-231 cells were grown in L-15 culture media. All the media were supplemented with 10% FBS and an antibiotic cocktail containing penicillin (5 mg/ml) and streptomycin (5 mg/ml) (GIBCO, Invitrogen, United Kingdom). HEK293 cells were kept in a humidified atmosphere of 95% O_2_ and 5% CO_2_ in a CO_2_ incubator at 37°C while MDA-MB-231 cells were kept in 100% O_2_ incubator at 37°C. The exponentially growing cultured cells were used for experiments in the present study.

### Determination of *in Vitro* Cytotoxicity of DEAE-Dextran

#### MTT Cytotoxicity Assay

*In vitro* studies included MTT cytotoxicity assay and trypan blue exclusion assay performed using MCF-7 as well as MDA-MB-231 cell lines. For the MTT assay, briefly, respective cells were seeded at a concentration of 1 × 10^4^ cells in triplicate wells in a 96 well plate for each drug concentration. DEAE-Dextran and paclitaxel were added onto the adherent cells the following day. The corrected averages of proliferating cells were determined by subtracting the average reading of DMEM (background measurement) from the averages obtained for control or treatment conditions. The percentage of proliferating cells was determined relative to the number of control cells. Results are expressed as the average of five independent experiments (Ranjan and Pathak, 2016).

#### Trypan Blue Exclusion Assay

In the trypan blue exclusion assay, the cells were treated as earlier in the MTT cell proliferation assay. At the end of the incubation, cells were harvested and washed once with DPBS. Thereafter, 10 μl of cell suspension were mixed with 10 μl trypan blue dye. Subsequently, 10 μl of the sample was placed in the chambers of the counting slide. Live and dead cells were counted in an automated cell counter (Countess II automated cell counter, Thermo Fisher Scientific, United States). The percentage of cell death was calculated (% cell death = number of dead cells/total number of cells ×100) (Ranjan and Pathak, 2016).

### Determination of *in Vitro* ROS Scavenging Activity of DEAE-Dextran

Further, reactive oxygen species (ROS) activity was determined using DCFDA fluorescent probe in both MDA-MB-231 and HEK293 cells and recorded at 490 nm excitation and 530 nm emission. Initially, cell plating was carried out at a seeding density of 2 × 10^4^ cells in a 96 well plate. The cells were allowed to adhere for 24 h. DEAE-Dextran and paclitaxel (1 and 5 μM) treatment was given to the cells. In peroxide induced ROS; hydrogen peroxide (100 μM) was added after incubation with drug for 3 h. The fluorescent probe DCFH-DA was then added at a concentration of 25 μM and incubated in dark for 1 h. The fluorescence of DCF was recorded at above mentioned excitation and emission (Ranjan and Pathak, 2016).

### Evaluation of *in Vitro* Interferon Inducing Capacity of DEAE-Dextran

#### Gene Expression Studies for Various Types of Interferons

Gene expression studies for various interferons; α-interferon, β-interferon, and γ-interferon were performed from *in vitro* cell cultures of HEK293 and MDA-MB-231 cells. The forward and reverse primers for the various genes were synthesized (**Table 1**). Initially, RNA was isolated using Trizol^®^ reagent followed by cDNA synthesis using the Thermo Scientific RevertAid First Strand cDNA synthesis kit. Gradient PCR studies were then carried out for the various genes in order to determine the best available Tm for the primers synthesized. The PCR products were then qualitatively analyzed by gel electrophoresis using the gel documentation system and further analyzed against housekeeping gene GAPDH using ImageJ software.

**Table 1:** Gene sequences and properties.

Gene	Forward, reverse	GenBank accession number	Tm (°C)
VEGF	5′GGAGCAGAAAGCCCATGAAGTG	KT971352.1	55.6
	5′AAGGCTCACAGTGATTTTCTGGC		
PEA3	5′ATGCACCAATCAGCTGCTCC	XM_006247372.3	55.8
	5′GTCATAGGCACTGGAGTAAAGACAC		
NOTCH1	5′GTGTGTGAAAAGCCCGTGTC	NM_001105721.1	53.8
	5′GCACAAGGTTCTGGCAGTTG		
β-Interferon	5′AAAGAAGCAGCAATTTTCAG	NM_002176.3	58.9
	5′TTTCTCCAGTTTTTCTTCCA		
α-Interferon	5′ CTGTGTGATACAGGGGGTGG	M54886.1	63
	5′ ATCGTGTCATGGTCATAGCAG		
γ-Interferon	5′ TCGTTTTGGGTTCTCTTGGC	AM903379.1	66.6
	5′ TGCTTTGCGTTGGACATTCAA		
GAPDH	5′ATGTTCGTCATGGGTGTGAACCA	AF106860.2	70.3
	5′TGGCAGGTTTTTCTAGACGGCAG		


#### Measurement of β-Interferon Levels

β-Interferon released was measured in various treated and control cell supernatants in both HEK293 and MDA-MB-231 cells. The standards were diluted independently according to the instruction manual. Samples were injected in the following manner: (1) Blank well-Sample, anti INF-β antibody labeled with biotin and streptavidin-HRP was not added; chromogen reagent A and B, stop solution was added. (2) Standard solution wells: 50 μl standard and streptomycin-HRP 50 μl; (3) sample wells- 40 μl sample added and 10 μl IFN-β antibodies, 50 μl streptavidin-HRP. The plate was then covered and gently shaken to mix and incubated for 60 min at 37°C. Five times washing was carefully done followed by bloating the plate. Then, 50 μl chromogen reagent A and reagent B was added and shaken to mix and further incubated for 10 min at 37°C away from light for color development. 50 μl stop solution was then added to each well to stop the reaction. Taking blank as zero, absorbance of each well was measured at 450 nm.

### Anti-cancer Potential of DEAE-Dextran in *in Vivo* DMBA Induced Mammary Cancer Model

#### Experiment Animals

This study was carried out in accordance with the recommendations of CPCSEA guidelines. All experiments and protocols described in present study were approved by the Institutional Animal Ethics Committee (IAEC) of Institute of Pharmacy, Nirma University, Ahmedabad. 56 3–5 week old female SD rats were procured from Zydus Research Centre, Ahmedabad. Animals were housed in the animal house of Nirma University, Ahmedabad under controlled conditions of temperature 23 ± 2°C, relative humidity 55 ± 5%, and 12 h light and dark cycles.

#### Experimental Design and Protocol

Female SD rats were randomized into eight groups, respectively; control animals, positive control, control treated with DEAE-Dextran (100 mg/kg, p.o.), rats treated with DEAE-Dextran (100 mg/kg, p.o.), control treated with paclitaxel (30 mg/kg, p.o.), rats treated with paclitaxel (30 mg/kg, p.o.), control treated with β-interferon (3 μg/kg, i.m.), and rats treated with β-interferon (3 μg/kg, i.m.).

25 mg/kg DMBA was dissolved in a mixture of olive oil and saline in ratio 3:1 and administered subcutaneously in the right side mammary gland to the diseased animals. Simultaneously, the time of tumor incidence was recorded along with the tumor size. The drug treatment with DEAE-Dextran, paclitaxel, and β-interferon was started simultaneously after tumor detection, respectively. After 3 months, tumors were isolated and subjected to various biochemical parameters, histopathological studies, immunohistochemistry, and gene expression studies.

#### Tumor Regression Analysis

Tumor regression analysis was carried out by measuring the tumor volume weekly. Tumor volume was measured using the formula; Tumor volume (mm^3^) = 4/3π (1/4 (Tumor length + Tumor width))^3^.

#### Determination of Anti-oxidant Tumor Biomarker Activity

Tumors were dissected out and homogenized in phosphate buffer saline (PBS) for the determination of various biochemical parameters.

Various anti-oxidant parameters were analyzed from tumor homogenates. Superoxide dismutase (SOD) and catalase were analyzed by kinetic absorption method prescribed previously by Aebi and Misra, respectively (Variya et al., 2015). SOD was expressed as units/min/mg of protein and catalytic activity was expressed for as μmoles of H_2_O_2_ consumed/min/mg of protein and for (Variya et al., 2015). Glutathione (GSH) was measured by the method of Moron et al. (1979) and the results were expressed in terms of μg of GSH/mg of protein. Lactate dehydrogenase (LDH) and gamma glutamyl transferase (GGT) were evaluated using the respective accucare kit protocols.

#### Histopathological Studies

For histopathology studies, the breast tissue was fixed in 10% neutral formalin, embedded in paraffin, 5 μm sections were cut and stained with hematoxylin-eosin stain and also separate staining with Mayer’s mucicarmine was carried out to detect presence of mucinous carcinoma.

#### Measurement of β-Interferon Levels in DMBA Induced Mammary Cancer Model

β-interferon levels were measured in the serum samples as well as mammary gland homogenates of various treatment and control groups as per the protocol described above.

#### Immunohistochemistry Studies for Various Tumor Marker Proteins

Immunohistochemistry staining for ER, PR, HER2, CD31, ki67, CK5/6, bcl2, p63, and p53 were performed. 5 μm sections were subjected to a serial process of dehydration and hydration for receptor activation followed by antibody staining (dilution 1:400) and incubation for 1 h. This was preceded by blocking and secondary antibody incubation for another 1 h. Finally, DAB chromogen was added for detection of the stain and hematoxylin counter staining performed. The slides were then mounted and observed for immunohistochemical staining using Olympus microscope. The immunoreaction was determined by the integrated optical density measurement.

#### *In Vivo* Protein Expression Studies

Protein expression studies were carried out by western blot technique for various proteins; PCNA, β-catenin, and E-cadherin along with housekeeping gene β-actin. Proteins from tumor samples were homogenized in PBS containing protease inhibitor followed by determination of protein concentration using BCA protein assay kit. Uniform protein samples were loaded into the wells of the stacking gel and run until the bromophenol reached the bottom of the gel. Gels were then transferred to nitrocellulose paper at 4°C at 30–40 volts for 3 h. The membrane was then blocked in 5% BSA prepared in Tween PBS, for 1 h at RT. Detection of the protein of interest was achieved by probing the nitrocellulose membrane with the primary antibody of interest. Primary antibodies used were PCNA, β-catenin, E-cadherin and β-actin, the latter used to ensure equal loading of samples. Molecular weight marker enabled the determination of protein sizes. The intensity of bands was then detected by ECL detector and analyzed using ImageJ software (Ranjan and Pathak, 2016).

### *In Vitro* Gene Expression Studies for VEGF, NOTCH1, and PEA3

Gene expression studies for VEGF, NOTCH1, PEA3, α-interferon, β-interferon, and γ-interferon were performed from *in vitro* cell cultures of HEK293 and MDA-MB-231 cells. The forward and reverse primers for the various genes were synthesized with the sequences as per **Table 1**. Initially, RNA was isolated using Trizol^®^ reagent followed by cDNA synthesis using the Thermo Scientific RevertAid First Strand cDNA synthesis kit. Gradient PCR studies were then carried out for the various genes in order to determine the best available Tm for the primers synthesized. The PCR products were then qualitatively analyzed by gel electrophoresis using the gel documentation system and further analyzed against housekeeping gene GAPDH using ImageJ software.

### Determination of Apoptosis by DEAE-Dextran

Further, flow cytometry was carried out to evaluate the apoptotic nature of DEAE-Dextran. MDA-MB-231 cells (1 × 10^5^) were seeded into a six well plate for 24 h. Cells were treated with DEAE-Dextran, β-interferon, and paclitaxel. Briefly, after 24 h post-treatment, 1 × 10^6^ cells were isolated in 1 × annexin binding buffer and treated with both fluorescein isothiocyanate-annexin V stain and propidium iodide. After 30 min incubation, the samples were subjected to analysis by flow cytometry (FACSAria 3, BD Biosciences, San Jose, CA, United States) (Ranjan and Pathak, 2016).

### Statistical Analysis

Statistical analysis results are represented as mean ± SEM. Statistical analysis was performed using Graphpad prism 5 statistical software. Statistical differences between the means of various groups were evaluated using one way analysis of variance (ANOVA) followed by Tukey’s test. Data were considered statistically significant at *P* < 0.05.

## Results and Discussion

### *In Vitro* Cytotoxic Activity of DEAE-Dextran

**FIGURE 1:**
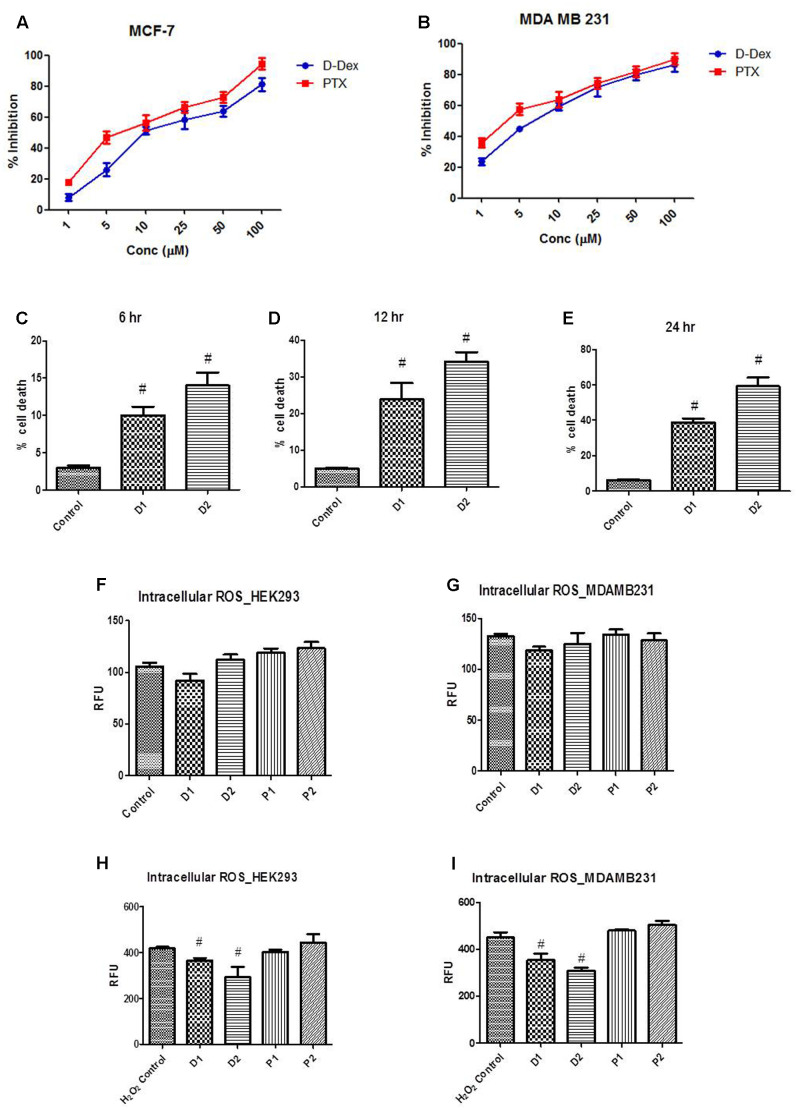
Determination of LD_50_ of diethylaminoethyl dextran (DEAE-Dextran) by MTT cell proliferation assay; **(A)** % cell proliferation inhibition in MCF-7 cell line and **(B)** % cell proliferation inhibition in MDA-MB-231 cell line. Trypan blue cell viability assay; **(C)** The % cell death at 6 h post-treatment **(D)** % cell death 12 h post-treatment **(E)** % cell death 24 h post-treatment. Reactive oxygen species (ROS) measurement post DEAE-Dextran treatment; **(F)** ROS in HEK293 cells without peroxide pre-incubation, **(G)** ROS in MDA-MB-231 cells without peroxide pre-incubation, **(H)** ROS measurement in HEK293 cells with hydrogen peroxide pre-incubation and **(I)** ROS measurement in MDA-MB-231 cells with hydrogen peroxide pre-incubation. ^#^ Significantly different from control (*P* < 0.05), Values expressed as Mean ± SEM. H_2_O_2_ Control – hydrogen peroxide (100 μM) treated cells, P1 – 1 μM paclitaxel, P2 – 5 μM paclitaxel, D1 – 1 μM DEAE-Dextran, D2 – 5 μM DEAE-Dextran.

MTT assay is used as a sensitive, quantitative, and reliable colorimetric assay to measure viability, proliferation, and activation of cells. The MTT assay was performed to determine the IC_50_ for DEAE-Dextran in MCF-7 and MDA-MB-231 cell lines using paclitaxel as a standard anti-cancer drug for breast cancer. It was calculated that the IC_50_ values of DEAE-Dextran and Paclitaxel were found to be 9.8 and 8.073 μM, respectively. Further, we determined the same in the triple negative breast cancer cell line MDA-MB-231 and the IC_50_ for DEAE-Dextran and paclitaxel were found to be 4.9 and 4.2 μM, respectively. The results show excellent potency of DEAE-Dextran against the triple negative cell line MDA-MB-231 as compared to paclitaxel as a standard drug for the disease management while results on luminal A MCF-7 cell line show that DEAE-Dextran exhibits significant cytotoxicity (**Figures 1A,B**).

Trypan blue exclusion assay in MDA-MB-231 cells showed significant increase in % cell death in D1 (1 μM) and D2 (5 μM) as compared to the control cells without treatment. The linearity in % cell death was found to be significantly increased during a duration of 6, 12, and 24 h of treatment with significantly higher % cell death in 5 μM DEAE-Dextran up to 69% compared to control cells at 6% cell death. The results complied with the MTT assay data confirming the cytotoxicity of DEAE-Dextran in various breast cancer cell lines (**Figures 1C–E**).

### ROS Scavenging Potential of DEAE-Dextran

Reactive oxygen species are chemically reactive molecules which have essential functions in the body (Boonstra and Post, 2004). A moderate elevation in the ROS promotes cell proliferation and differentiation, whereas excessive amounts lead to oxidative damage to lipids, proteins, and DNA. Hence maintaining the ROS homeostasis is crucial for normal cell growth and survival (Boonstra and Post, 2004). Initially, the ROS activity was determined without pre-incubation with hydrogen peroxide and it was observed that there was no significant difference in the RFU in DEAE-Dextran and paclitaxel treated cells compared to untreated cells (**Figures 1F,G**). Later, after a 30 min pre-incubation with hydrogen peroxide, the cells were observed for ROS activity and it was observed that DEAE-Dextran at both 1 and 5 μM doses, exhibited significant reduction in ROS activity as compared to the untreated cells. However, paclitaxel was found to significantly enhance the ROS activity as compared to the untreated cells which was in line with the previously reported data (**Figures 1H,I**). Studies have reported the downregulation of ROS due to β-interferon in multiple sclerosis patients treated with β-interferon. The mechanism suggests that β-interferon renders NADPH oxidase (NOX) of cells less sensitive to the triggering agent of ROS. Also, paclitaxel has repeatedly proved to generate ROS in tumor cells through enhancement of NOX activity associated with plasma membranes (Kane et al., 1993; Alexandre et al., 2007).

### *In Vitro* Interferon Inducing Capacity of DEAE-Dextran

**FIGURE 2:**
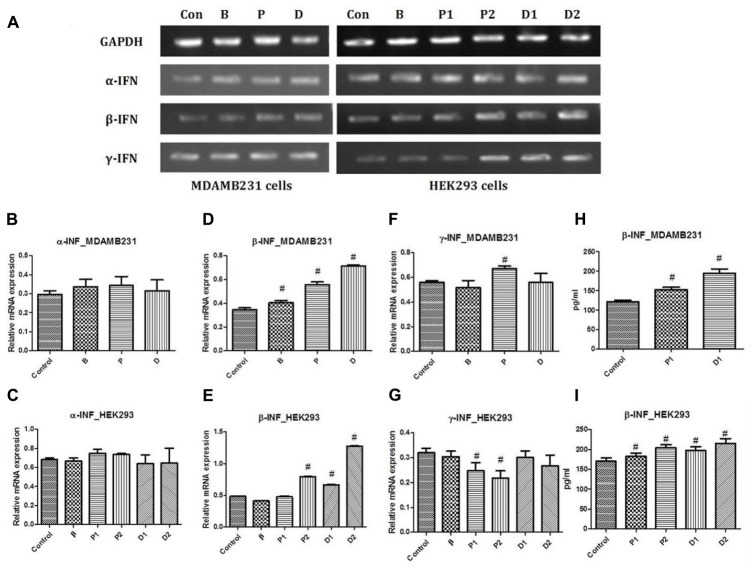
**(A)** Representative PCR bands in various treated cells, **(B)** α-interferon gene expression study in MDA-MB-231 cells, **(C)** α-interferon gene expression study in HEK293 cells, **(D)** β-interferon gene expression study in MDA-MB-231 cells, **(E)** β-interferon gene expression study in HEK293 cells, **(F)** γ-interferon gene expression study in MDA-MB-231 cells and **(G)** γ-interferon gene expression study in HEK293 cells. Determination of β-interferon release in various cell lines; **(H)** β-interferon release in MDA-MB-231 cell line and **(I)** β-interferon release in HEK293 cell line. ^#^ Significantly different from control (*P* < 0.05), Values expressed as Mean ± SEM. Control and con – untreated HEK293 and MDA-MB-231 cells, P1 and P – 1 μM paclitaxel, P2 – 5 μM paclitaxel, D1 and D – 1 μM DEAE-Dextran, D2 – 5 μM DEAE-Dextran, B and β – β-interferon treated cells.

PCR studies for α-interferon, β-interferon, and γ-interferon were conducted against GAPDH as the housekeeping gene. Interferon-α has been studied previously for its anti-proliferative and maximal anti-tumor activity *in vivo* pertaining to the modulation of expression of various oncogenes as well as increasing MHC I expression hence favoring immune recognition (Indraccolo et al., 2002). No alteration in the gene expression studies with α-interferon was observed in the treated cells as compared to the untreated cells. However, slight increase in the α-interferon expression was seen with paclitaxel but not significant enough to be declared a positive up regulatory gene for the same (**Figures 2A–C**).

Interferon-β is known to have a strong ability to inhibit tumor cell growth and induce apoptosis *in vitro* (Bakrania et al., 2018). Increasing evidence suggests that endogenous apoptosis inducers and cell growth regulators are important targets for effective cancer therapy. β-Interferon induces apoptosis in cancer cells basically by disrupting mitochondria and activating the caspase cascade, also, it is known as a potent inhibitor of proliferation of many cancer cell lines *in vitro* (Johns et al., 1992; Arai et al., 1997; Shimizu et al., 1998; Shinoura et al., 1998; Juang et al., 2004; Reed and Pellecchia, 2005; Reed, 2006; Matsuzuka et al., 2010). Gene expression studies revealed up-regulation of β-interferon in DEAE-Dextran and paclitaxel treated MDA-MB-231 cells which was relatively threefold more than the normal expression observed in the untreated cells (**Figures 2A,D,E**).

Interferon-γ has been studied to inhibit cell proliferation due to its ability to regulate several genes related to apoptosis or proliferation (Marth et al., 2004). It has been studied to have been secreted by a variety of tumor-infiltrating hematopoietic cells, and there is considerable experimental evidence suggesting that endogenous INF-γ plays a role in inhibiting tumor development, growth, and metastasis (Boehm et al., 1997; Redelman and Hunter, 2008). The transcriptional activity of interferon-γ remained unchanged during the treatment period with DEAE-Dextran and β-interferon. However, downregulation of interferon-γ was observed in the paclitaxel treated cells which was not significant enough to be concluded as a positive downregulation (**Figures 2A,F,G**).

The *in vitro* measurement of β-interferon released in HEK293 cell line capable of β-interferon production was used. A significant increase in β-interferon release was observed in 1 and 5 μM DEAE-Dextran treated cells after 24 h as compared to untreated HEK293 cells. Also, it was observed that paclitaxel exhibits β-interferon release in HEK293 cells which was significantly less than that of DEAE-Dextran at the respective treatment doses. Further, we determined the β-interferon release in MDA-MB-231 cells and observed a significant increase in β-interferon release in both paclitaxel and DEAE-Dextran treated cells with significantly higher β-interferon levels in DEAE-Dextran treated cells after 24 h treatment (**Figures 2H,I**).

### Anti-tumor Activity of DEAE-Dextran in DMBA Induced Mammary Cancer Animal Model

**FIGURE 3:**
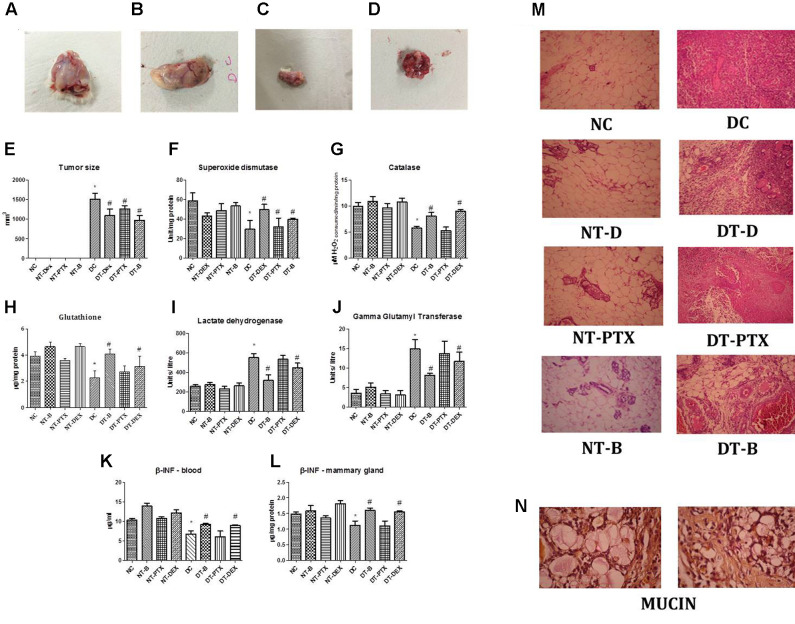
Representative photograph of tumor regression analysis in DMBA induced mammary cancer animal model; **(A)** positive control mammary gland, **(B)** rats treated with paclitaxel, **(C)** rats treated with β-interferon and **(D)** rats treated with DEAE-Dextran. **(E)** Graphical representation of tumor size. Biochemical estimations in DMBA induced mammary cancer model; **(F)** superoxide levels, **(G)** catalase levels, **(H)** glutathione (GSH) levels, **(I)** lactate dehydrogenase (LDH) levels, **(J)** gamma glutamyl transferase (GGT) levels, **(K)** β-interferon levels in blood and **(L)** β-interferon levels in mammary gland. **(M)** Histopathological studies in DMBA induced mammary cancer model (HE staining), magnification X250. **(N)** Histopathological studies with Mayer’s mucicarmine stain in positive control sections, magnification X400. ^∗^Significantly different from control animals (*P* < 0.05), ^#^Significantly different from positive control (*P* < 0.05), each group consists of six animals, Values expressed as Mean ± SEM. NC – control rats, DC – positive control, DT-Dex – 100 mg/kg DEAE-Dextran treated, DT-PTX – 30 mg/kg paclitaxel treated, DT-B – β-interferon treated, NT-Dex – normal treated with 100 mg/kg DEAE-Dextran, NT-PTX – normal treated with 30 mg/kg paclitaxel and NT-B – normal treated with β-interferon.

The region of DMBA administration at the mammary gland was regularly observed for any significant morphological changes. On day 22, a minimal tumor was observed which gradually increased in size reaching an optimal size within the next 9 weeks (**Figures 3A–E**).

Various anti-oxidant parameters and tumor biomarkers were evaluated in order to determine the activity of DEAE-Dextran on the same. SOD and catalase, primary anti-oxidants were evaluated for since they play a greater role in the protection of cells against oxidative damage induced by DMBA. SOD levels also enable protection of the biological integrity of cells and tissues against harmful effects of superoxide free radicals while catalase has shown to reduce the chromosomal aberrations delaying the onset of neoplastic transformation in previous reported studies (Jones et al., 1985; El-Bayoumy et al., 2000; Lakshmi and Subramanian, 2014). Results manifest decreased SOD and catalase levels in positive control as compared to control group. The animals treated with DEAE-Dextran, β-interferon, and paclitaxel showed significant increase in both anti-oxidant levels as compared to the diseased animals amongst which the order of increased levels was paclitaxel < DEAE-Dextran < β-interferon. The normal treated animals with DEAE-Dextran, β-interferon, and paclitaxel also showed significant increase in SOD and catalase levels compared to the control group (**Figures 3F,G**). As another preliminary anti-oxidant, GSH levels were evaluated for in the DEAE-Dextran treated animals since GSH reacts with free radicals acting as a crucial substrate for GPx and GST, which takes place in the cellular defense mechanisms against intermediate oxygenated products of metabolism. GSH also protects thiol groups in protein from oxidation functioning as a redox buffer and serves as a reservoir for cysteine (Moron et al., 1979). The results showed a significant decrease in GSH levels in positive control animals as compared to control group. The animals treated with DEAE-Dextran, β-interferon, and paclitaxel showed improvement in GSH levels (**Figure 3H**). Overall, elevated SOD, catalase and GSH levels in DEAE-Dextran treated animals implying its anti-oxidant potential in combating tumorigenesis.

As a measure of anaerobic conditions during tumor hypoxia, LDH enzyme activity was measured since it is coupled with the oxidation of NADH to NAD^+^ and normal tissues usually pertain to a distinct LDH activity pattern which increases during hypoxia as in tumor cells acting as a link between glycolysis and Warburg effect (Chatterton et al., 2002; Miao et al., 2013). LDH levels significantly increased in positive control animals as compared to control animals. Treated group of animals with DEAE-Dextran, β-interferon, and paclitaxel showed significant decrease in LDH levels as compared to positive control animals showing the restoration of anaerobic conditions (**Figure 3I**).

Also, GSH/GGT-dependent pro-oxidant reaction has been found to modulate the transduction of proliferative/apoptotic signals. The enzyme GGT uniquely enables GSH catabolism by hydrolysing the gamma glutamyl bond between glutamate and cysteine. It is chiefly present on the external surface of most cells but particularly studied as a biomarker of hepatic dysfunction. The hydrogen peroxide produced by GGT produces an anti-apoptotic activity in carcinoma cells. Studies have reported that increased levels of GGT led to higher rates of DNA damage and hence more oxidative bases (Corti et al., 2009; Fentiman and Allen, 2010). GGT levels were significantly increased in positive control animals as compared to control animals. Animals treated with DEAE-Dextran, β-interferon, and paclitaxel have shown to decrease GGT levels significantly as compared to positive control animals which indicates lesser chances of DNA damage and oxidative stress (**Figure 3J**).

During histopathological studies, hematoxylin-eosin and mucin staining was performed. HE staining of diseased animals showed significant increase in inflammation, tissue damage as well as hyperplasia as compared to control animals which maintained the intact alveolar structure of the mammary gland. The DEAE-Dextran, paclitaxel, and β-interferon treated animals showed decreased signs of inflammation and leukocyte infiltration as well as tumor differentiation which indicated an improvement in retaining the normal architecture of the mammary gland (**Figure 3M**). Mucin staining using Mayer’s mucicarmine showed presence of mucin as a pink froth in diseased tissues which was absent in the normal group. The treatment with DEAE-Dextran, β-interferon, and paclitaxel did not produce any significant decrease in mucin levels as compared to control animal tissues (**Figure 3N**). Presence of mucin signifies a rare sub-class of breast cancer known as mucinous carcinoma. Studies have suggested that mucinous carcinoma is usually negative for HER2 overexpression and further suggesting that HER2 is rarely involved in tumorigenesis of mucinous carcinoma. Further, mucin levels have been reported as an indication of tumor aggressiveness in human breast cancer (Hsu and Shaw, 2005).

### Enhancement of β-Interferon Levels in Serum and Mammary Gland with DEAE-Dextran Treatment

As per the previously discussed results of the *in vitro* interferon inducing capacity, we further analyzed the blood serum and mammary gland β-interferon levels in the *in vivo* DMBA induced mammary cancer model. β-Interferon has shown to exhibit a pronounced anti-proliferative activity in breast cancer cell lines. Its mechanism of action has been studied as prolonged intermitotic interval in cultured human fibroblasts and the arrest in G_0_–G_1_ phase of the cell cycle. Pertaining to hormonal levels, it has found to produce an increase of hormone receptors for ER and PR in tumor biopsy samples (Sica et al., 1987). Topotecan, a topoisomerase I-targeting drug, with well-defined mechanism of action, acts through elevated β-interferon expression in breast cancer cells (Wan et al., 2012). Several reports pertaining to β-interferon have shown its role as a tumor cell growth inhibitor and apoptosis inducer acting through Jak-Stat1 intracellular signaling pathways, including immunomodulatory and anti-angiogenic effects, *in vitro* and *in vivo* (Matsuzuka et al., 2010; Yi et al., 2014).

Blood serum β-interferon levels were measured by ELISA and observed to be downregulated in positive control group as compared to control animals. However, rats treated with DEAE-Dextran and paclitaxel showed to significantly increase β-interferon levels whereas β-interferon treated did not produce any significant change in β-interferon amounts compared to positive control animals. Further, normal treated with DEAE-Dextran and paclitaxel showed slight increase in β-interferon levels while β-interferon treated showed no significant effect on β-interferon levels compared to control animals (**Figure 3K**). β-Interferon levels in mammary gland homogenate showed similar results whereby there was a significant decrease in β-interferon levels in the diseased tissue homogenates as compared to control animals. Similar to the blood serum analysis, the treated animals with DEAE-Dextran and paclitaxel showed significant increase in β-interferon levels while β-interferon treated did not produce any significant change as compared to positive control animals. The normal treated with DEAE-Dextran and paclitaxel also showed significant increase in β-interferon levels (**Figure 3L**). This quantitative analysis of β-interferon levels proves the induction of β-interferon release by DEAE-Dextran as well as paclitaxel. Previous studies have proven β-interferon induction by various chemotherapeutic agents such as paclitaxel further leading to elevated MHC I expression in breast cancer cells hence suggesting that elevated tumor antigen presentation through β-interferon autocrine/paracrine signaling could represent a common mechanism underlying tumor immune sensitization by cancer therapeutics (Wan et al., 2012). Similarly, at this juncture we can hypothesize that DEAE-Dextran could act as an anti-cancer agent following the same mechanism of MHC I expression, however, it remains unproved till exhaustive research studies.

### DMBA Induced Mammary Cancer Exhibits Double Negative Breast Cancer Pertaining to ER, PR, and HER2 Irrespective to DEAE-Dextran Treatment

**FIGURE 4:**
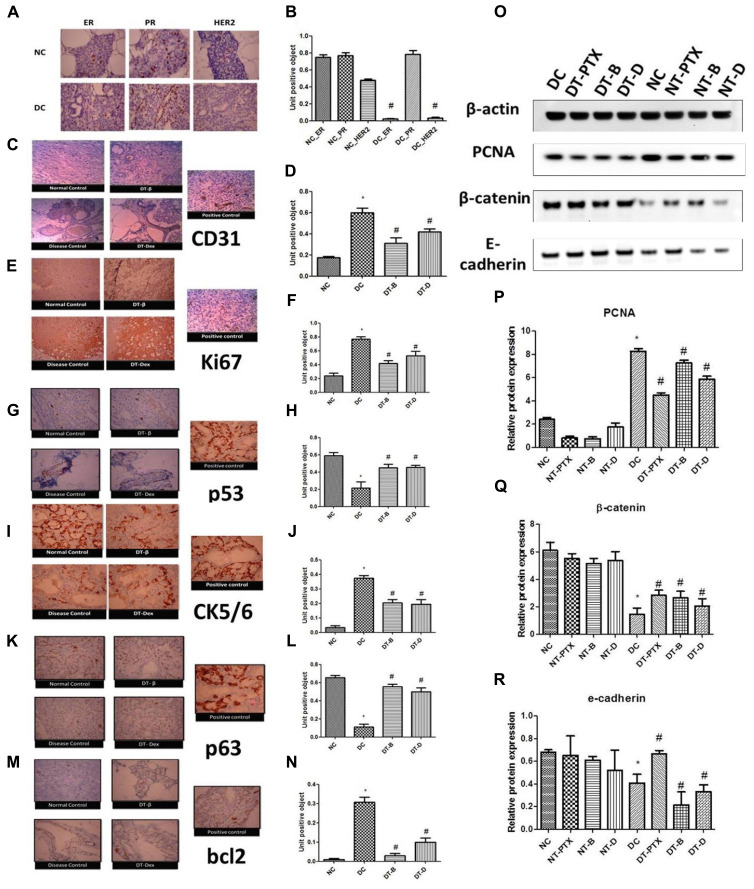
**(A,B)** Immunohistochemistry studies for ER, PR, and HER2 in DMBA induced mammary gland model. NC – control rats, DC – positive control, ER – estrogen antibody staining, PR – progesterone antibody staining, HER2 – HER2 antibody staining, magnification X100. **(C,D)** Immunohistochemistry studies of CD31 in DMBA induced mammary gland model. Positive control of liver section was used, magnification X100. **(E,F)** Immunohistochemistry studies of ki67 in DMBA induced mammary gland model. Positive control of breast carcinoma was used, magnification X100. **(G,H)** Immunohistochemistry studies of p53 in DMBA induced mammary cancer model. Positive control breast cancer sections were used, magnification X100. **(I,J)** Immunohistochemistry studies of CK5/6 in DMBA induced mammary cancer model. Positive control of lung squamous cell carcinoma slide was used, magnification X100. **(K,L)** Immunohistochemistry studies of p63 in DMBA induced mammary cancer model. Positive control as breast cancer section was used, magnification X100. **(M,N)** Immunohistochemistry studies of bcl2 in DMBA induced mammary gland model. Positive control of tonsil section was used. Control animals, positive control, DT-B – rats treated with β-interferon, DT-Dex – rats treated with DEAE-Dextran, magnification X100. Determination of protein expression by western blot analysis; **(O)** representative western blot bands, **(P)** determination of PCNA protein expression, **(Q)** determination of β-catenin protein expression, and **(R)** determination of E-cadherin protein expression. ^∗^Significantly different from control animals (*P* < 0.05), ^#^ Significantly different from positive control (*P* < 0.05), each group consists of six animals, Values expressed as Mean ± SEM. NC – Control animals, DC – positive control, DT-D – 100 mg/kg DEAE-Dextran treated, DT-PTX – 30 mg/kg paclitaxel treated, DT-B – β-interferon treated, NT-D100 – normal treated with 100 mg/kg DEAE-Dextran, NT-PTX – normal treated with 30 mg/kg paclitaxel and NT-B – normal treated with β-interferon.

Immunohistochemistry for ER, PR, and HER2 was performed which showed negative ER and HER2 levels and positive PR levels in positive control animals while all the three were found significantly present in control animals. Treatment with DEAE-Dextran, β-interferon, and paclitaxel did not produce any significant change in the ER, PR, and HER2 levels and maintained negative ER and HER2 and positive PR. Similarly, normal treated tissues also maintained all the three proteins ER, PR, and HER2 (**Figures 4A,B**). Previous studies have reported that although hormone independent tumors do exist such as triple negative breast cancer, usually, in chemically induced mammary carcinoma, progesterone is evidently present due to its function for the differentiation of alveolar structures and enhancement of proliferation in the mammary gland. Similarly, HER2 negative expression is evident due to the positive mucinous carcinoma as discussed above and studies proven they rarely co-exist (Chow et al., 2003). Henceforth, it can be justified that DEAE-Dextran does not act on the hormonal mechanism and therefore would be a more promising option for triple negative breast cancer which lacks ER, PR, and HER2.

### Anti-angiogenic Potential of DEAE-Dextran: Downregulation of CD31

CD31 is a member of the immunoglobulin superfamily and a surface molecule mediating homo- and heterotypic interactions which control leukocyte trafficking through the endothelial layer. Also, it rules a complex signaling pathway, partially intertwined with the pathway controlled by integrins (Newman et al., 1990; Lu et al., 1996). However, far, CD31 expression had never been demonstrated in breast cancer cells. Thus, work had been experimented for the observation of CD31 expression in ductal carcinoma *in situ* in breast cancer due to the role of angiogenesis in the evident type of cancer. It has attracted attention of basic research due to its involvement in multi-step adhesion processes and wide use in breast pathology as a marker of angiogenesis (Newman et al., 1990). IHC for CD31 was performed and it showed significantly increased CD31 expression in positive control tissues as compared to control animal tissues. However, treated tissues showed significant decrease in CD31 expression indicative of anti-angiogenic potential of DEAE-Dextran in the *in vivo* study (**Figures 4C,D**).

### DEAE-Dextran Exhibits Anti-proliferative Activity

Several markers exist for the determination of the anti-proliferative activity of novel molecules. We have henceforth utilized ki67, p53, and PCNA for evaluating the effect of DEAE-Dextran on cell proliferation preceding the preliminary *in vitro* MTT and trypan blue assays. Ki67 is a nuclear protein expressed in G_1_, G_2_, S, and M phases but not expressed in G_0_; as such, its expression can be used as an indicator of actively proliferating cells (Gerdes et al., 1984). In the present study, ki67 expression showed increased nuclear staining in positive control animal tissues as compared to control animal tissues. However, significant decrease in ki67 expression was observed in treated tissues with DEAE-Dextran, β-interferon, and paclitaxel as compared to diseased tissue signifying its anti-proliferative action (**Figures 4E,F**).

Similarly, P53 protein (also known as TP53), the guardian of stability and genome integrity, acts to circumvent cell proliferation due to DNA damage as well as activation of apoptosis in case of unrepairable damage (Lakhani et al., 2002). Several studies have indicated its function as a tumor suppressor gene conferring to suppress oncogene-induced transformation, proliferation, and *in vivo* tumorigenic capacity of cell lines (Finlay et al., 1989; Baker et al., 1990; Chen et al., 1990). Under physiological conditions, p53 has a very short half-life measured in minutes, however, when exposed to mutagenic agents, p53 is stabilized and accumulated within the nucleus. This accumulated p53 binds to DNA and causes cells to arrest in G_1_ phase. However, inactivation of p53 is most common by missense mutations and these abnormal proteins fail to bind to DNA having remarkably a long half-life, due to which they can readily be detected in tumor cells by IHC. Indeed, p53 proteins detected in this manner are almost always associated with mutations most of which contribute to the malignant transformation of normal cells (Frebourg and Friend, 1993; Myoung et al., 2001). Our results confirm negative p53 staining in control animal tissues indicating absence of mutations. However, the positive control samples showed excessive p53 staining which implies the highly expressed p53 in the nucleus which is due to the missense mutation as discussed previously (**Figures 4G,H**). The DEAE-Dextran, β-interferon, and paclitaxel treated samples indicated significantly reduced p53 protein which showed the benefit of treatment in reversing the mutation or in preventing mutations from occurring at the first incidence.

Finally, western blot analysis for Proliferating cell nuclear antigen (PCNA) was carried out to confirm the anti-proliferative property of DEAE-Dextran. It is a 36-kD non-histone nuclear polypeptide expressed primarily in S-phase cycling cells. In normal human cells, PCNA exists in a complex with a cyclin, cyclin-dependent protein kinase (CDK) and p21 induced by p53 (Xiong et al., 1992; Sakai et al., 1998). It has been identified as an auxiliary protein of DNA polymerase delta. Its distribution increases through G_1_, peaks at the G_1_/S-phase, decreases through G_2_ and reaches undetectable levels in M-phase and quiescent cells (Schönborn et al., 1994). PCNA is considered an indispensable protein for cell proliferation with the capability to function as a sliding clamp encircling the DNA double helix (Matunis, 2002; Maga and Hübscher, 2003; Moldovan et al., 2007; Zhao et al., 2014). Previous reports have discussed that PCNA is phosphorylated at the tyrosine residue 211, resulting in stabilization of PCNA on chromatin in breast cancer cells overexpressing EGFR. Results demonstrate significantly high PCNA expressions in positive control as compared to control animals. However, significantly decreased expressions were evident in the DEAE-Dextran, paclitaxel, and β-interferon treated proteins indicative of its anti-proliferative activity (**Figures 4O,P**).

### Treatment with DEAE-Dextran Upregulates Biomarkers CK5/6 and p63

CK 5/6 is a specific high molecular weight cytokeratin expressed in myoepithelial cells in normal breast tissue. Benign ductal epithelial hyperplasia contains CK 5/6 expression while malignant proliferation lacks the same. Accessing CK 5/6 expression minimizes the false-negative and false-positive diagnoses (Nofech-Mozes et al., 2008; Al-Maghraby et al., 2012). For CK 5/6, cytoplasmic staining was considered positive. For cell clusters, up to 10 epithelial clusters were graded on each slide for the presence of cytoplasmic staining, and the percentage of cell clusters containing CK 5/6 positive cells were scored. Highly positive staining was observed in the control rats as well as the normal treated samples, however, the positive control contained the least percentage of cytoplasmic stained cells implying minimal presence of CK 5/6. The treatment sections with DEAE-Dextran, β-interferon, and paclitaxel possessed increased cytoplasmic stains which conclude the upregulation of CK 5/6 implying healthy normal breast cells after treatment (**Figures 4I,J**).

P63 is another potentially useful marker for myoepithelial cells for distinguishing invasive from non-invasive breast lesions on histologic sections (Al-Maghraby et al., 2012). For p63, only nuclear staining was considered to be positive while cytoplasmic staining was counted as negative. Results obtained suggest that p63 was exclusively expressed in the myoepithelial cells of the control rat sections, partially expressed in the DEAE-Dextran, β-interferon, and paclitaxel treated samples and rarely expressed in the positive control sample indicating non-invasive carcinoma of the mammary gland (**Figures 4K,L**).

### DEAE-Dextran Improves Cell–Cell Adhesion in Breast Cells

In order to access the cell–cell adhesion activity of DEAE-Dextran, we evaluated the protein expression studies of β-catenin and E-cadherin. β-Catenin, a multifunctional protein, is located to the intracellular side of the cytoplasmic membrane playing a crucial role in cell-to-cell adhesion by linking cadherins to the actin cytoskeleton and with a central role in transcriptional regulation in the Wnt signaling pathway (Geyer et al., 2011). β-Catenin dysfunction has often been reported in various studies (Tetsu and McCormick, 1999; Lin et al., 2000). Results obtained show low β-catenin expressions in positive control as compared to control animals. Similarly, rats treated with DEAE-Dextran, paclitaxel, and β-interferon demonstrated significantly higher expression than the positive control which proves the presence of a well-differentiated tumor network corresponding to cell–cell adhesions (**Figures 4O,Q**).

Further, E-cadherin expressions were monitored in the *in vivo* mammary gland protein samples. E-cadherin, a120 kDa protein, also known as uvomorulin, forms the key functional component of adherens junctions between epithelial cells (Wijnhoven et al., 2000). Normal E-cadherin expression and function are essential for the induction and maintenance of polarized and differentiated epithelia during embryonic development. E-cadherin-catenin complex is a basic requirement for the maintenance of the normal intercellular adhesion. Also, it has been proposed that in cancer, E-cadherin functions as an invasion suppressor molecule such that its loss permits invasion of adjacent normal tissues. Generally, E-cadherin and β-catenin are strongly expressed in well-differentiated tumors which maintain their cell adhesiveness and are less invasive, however, they are reduced in poorly differentiated tumors which have lost their cell–cell adhesion and show strong invasive behavior (Takeichi, 1991; Shiozaki et al., 1996). E-cadherin was negatively expressed in the positive control as compared to control animals. However, paclitaxel exhibited increased E-cadherin expression in treated while DEAE-Dextran and β-interferon did not show any relevant expression levels to bring out a decisive conclusion that they confine to improving the cell–cell adhesion as well as show that the tumor might be poorly differentiated (**Figures 4O,R**).

### DEAE-Dextran Acts via Angiogenesis Pathway through Inhibition of VEGF and NOTCH1

**FIGURE 5:**
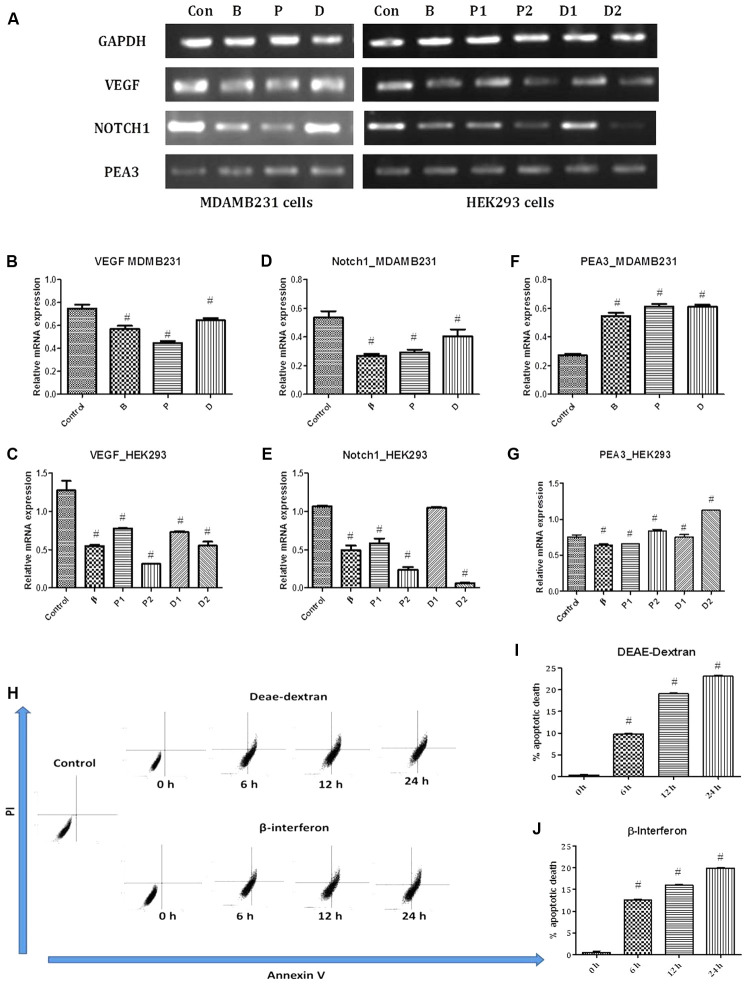
**(A)** PCR bands in various treated cells. Con – vehicle control cells, B – β-interferon treated cells, P1 – 1 μM paclitaxel treated cells, P2 – 5 μM paclitaxel treated cells, D1 – 1 μM DEAE-Dextran treated cells, and D2 – 5 μM DEAE-Dextran treated cells. **(B)** VEGF gene expression study in MDA-MB-231 cells, **(C)** VEGF gene expression study in HEK293 cells, **(D)** NOTCH1 gene expression study in MDA-MB-231 cells, **(E)** NOTCH1 gene expression study in HEK293 cells, **(F)** PEA3 gene expression study in MDA-MB-231 cells, and **(G)** PEA3 gene expression study in HEK293 cells. ^#^ Significantly different from control (*P* < 0.05), Values expressed as Mean ± SEM. Control – untreated HEK293 and MDA-MB-231 cells, P1 and P – 1 μM paclitaxel, P2 – 5 μM paclitaxel, D1 and D – 1 μM DEAE-Dextran, D2 – 5 μM DEAE-Dextran, B and β – β-interferon treated cells. Flow cytometric analysis for determination of apoptosis; **(H)** FACS analysis data of PI vs Annexin V in various treated samples up to a duration of 24 h, **(I)** % apoptotic death over-time post-treatment with DEAE-Dextran, and **(J)** % apoptotic death over-time post-treatment with β-interferon. ^#^ Significantly different from control cells (*P* < 0.05), Values expressed as Mean ± SEM. 0 h – control cells at 0 h treatment with DEAE-Dextran or β-interferon, respectively, 6, 12, 24 h – 6, 12, and 24 h treated cells, respectively.

Vascular endothelial growth factor (VEGF) is a well-known hypoxia-induced stimulator of endothelial cell growth and angiogenesis, which is also up-regulated and down-regulated by various hormones and cytokines (Ferrara and Davis-Smyth, 1997). Laboratory and clinical evidence supports the central role of angiogenesis in the progression of breast cancer and amongst the multiple angiogenic factors; the 121-amino acid isoform of VEGF predominates (Ferrara and Davis-Smyth, 1997). VEGF stimulates endothelial proliferation and migration, inhibits endothelial apoptosis, induces proteinases that remodel the extracellular matrix, increases vascular permeability and vasodilatation, and inhibits antigen-presenting dendritic cells. Tumor cells have been reported to contain high levels of VEGF mRNA in all types of breast cancer (Bando et al., 2005; Verrax et al., 2011). Results obtained showed strong VEGF expression in untreated MDA-MB-231 cells and in contrast, low labeling for receptor mRNA was detected in the DEAE-Dextran, paclitaxel, and β-interferon treated cells with least expression in paclitaxel treated MDA-MB-231 cells (**Figures 5A–C**). The reduced expressions in DEAE-Dextran and β-interferon treated cells was specifically due to the presence of β-interferon indirectly or directly, respectively. Interferons have been the first endogenous antiangiogenic regulators identified and studies have shown that human leukocyte INF (INF α/β) inhibited capillary endothelial cell motility *in vitro* (Brouty-Boyé and Zetter, 1980; Sidky and Borden, 1987). Studies have shown the downregulation of VEGF by β-interferon which has been re-observed in our results (Yoshida et al., 1994). Previous reports have discovered a new and unexpected additional characteristic of paclitaxel – its antiangiogenic activity. Although the anti-vascular effects of tubulin-binding agents have been previously reviewed, paclitaxel has shown potent anti-angiogenic effects in numerous *in vitro* and *in vivo* models with the molecular mechanism pertaining to VEGF downregulation as well as reduced IHC expression of CD31 which is also an angiogenic biomarker (Bocci et al., 2013). Hence, it can further be hypothesized that DEAE-Dextran exhibits an anti-angiogenic effect on the tumor microenvironment eventually leading to an anti-cancer effect.

Further, we also evaluated for the effect of DEAE-Dextran on NOTCH1 expression due to its central role in angiogenesis. Among NOTCH receptors with potential roles in breast carcinogenesis, NOTCH1 (MW 272kDa) is relatively the best studied (Diévart et al., 1999; Stylianou et al., 2006). The importance of NOTCH1 in carcinogenesis was first observed in transgenic mice generated by the mouse mammary tumor virus which can spontaneously develop oligoclonal CD4 CD8 T-cell tumors. NOTCH1 was further found to be a mediator of oncogenic Ras which occurs in early breast cancer playing a central role in breast cancer (Weijzen et al., 2002; Reedijk et al., 2005). Moreover, NOTCH1 and JAG1 are co-upregulated in breast cancer cell lines as well as endothelial cells suggesting its role in angiogenesis as well (Weijzen et al., 2002). In the MDA-MB-231 cells, there was evident suppression observed in all the treated cells with DEAE-Dextran, paclitaxel, and β-interferon compared to the untreated MDA-MB-231 cells which possessed an exceptional high expression of NOTCH1 gene (**Figures 5A,D,E**). VEGF was first shown to act upstream of NOTCH1 in determining arterial cell fate in vascular development and was demonstrated to increase notch expression. Further studies have established that VEGF regulates the expression of notch signaling components (Ferrara et al., 2003). This justifies the reduced expression of NOTCH1 in DEAE-Dextran, paclitaxel, and β-interferon treated cells which as discussed above led to decrease in VEGF mRNA levels. Additionally, rare studies have also confirmed the down-regulation of NOTCH1 by paclitaxel, which is as a result of the induction of NF-kB, further leading to induction of cell apoptosis. This study confirms dual mechanistic approach of DEAE-Dextran as a VEGF and NOTCH1 inhibitor which could be a potential target in paclitaxel resistance patients.

### DEAE-Dextran, As Anti-angiogenic Agent, Has No Correlation to the PEA3 Factor

We carried out Polyomavirus enhancer activator 3 (PEA3/E1AF/ETV4) expression study in order to identify whether DEAE-Dextran has any activity related to the ETS transcription factor. PEA3 is overexpressed in metastatic breast carcinomas, particularly triple negative breast tumors. PEA3 is a transcriptional activator of NOTCH1 and NOTCH4 and a repressor of NOTCH2 in MDA-MB-231 cells and its mediated NOTCH1 transcription is AP1 independent. PEA3 and/or NOTCH signaling are essential for proliferation, survival, and tumor growth of MDA-MB-231 cells (Clementz et al., 2011). Studies have indicated that exogenous PEA3 could activate VEGF promoter activity, suggesting that PEA3 played a role in regulating VEGF transcription (Hua et al., 2009). Similarly, transcription studies have suggested negative regulation of fibroblast growth factor 10 (FGF-10) by PEA3 proving its overexpression as anti-angiogenic (Chioni and Grose, 2009). Gene expression studies showed non-significant up-regulation of PEA3 in the DEAE-Dextran, β-interferon, and paclitaxel treated cells hence could not be considered as a positively regulated gene for the respective treatment (**Figures 5A,F,G**). It can therefore be predicted that DEAE-Dextran does not act through the PEA3 transcription for the activation of VEGF and NOTCH1 inhibition, hence, more significant mechanistic approach still remains undefined.

### Apoptosis: Another Mechanism Responsible for the Anti-tumor Activity of DEAE-Dextran

In order to determine whether DEAE-Dextran exhibits apoptosis, we carried out protein expression studies of the anti-apoptotic protein widely used- Bcl2 and flow cytometry analysis for the confirmation of apoptosis in triple negative breast cancer. B-cell lymphoma 2 protein (Bcl2) is an anti-apoptotic protein with conjoint anti-proliferative effect influencing cell-cycle entry and a powerful favorable prognostic breast tumor marker (Hockenbery et al., 1990; Pietenpol et al., 1994; O’Reilly et al., 1996; Huang et al., 1997; Greider et al., 2002; Callagy et al., 2006; Zinkel et al., 2006; Dawson et al., 2010; Ali et al., 2012). Although Bcl2 expression has been associated with ER status, it defines good prognostic subgroups in both ER-positive and ER-negative tumors (Dawson et al., 2010). The tumorigenic potential of Bcl2 was first described by Tsujimoto et al. (1984) and since this discovery, overexpression of Bcl2 has been identified in various tumors including breast tumor (Tsujimoto et al., 1984; Pardo et al., 2002). Increased levels of Bcl2 also disrupt the balance with various members of the Bcl2 family, including expressions of pro-apoptotic genes (Dawson et al., 2010). In the present study, results manifest rare Bcl2 expressed in the control rat samples which is converse to the positive control possessing highly expressed Bcl2 staining. The DEAE-Dextran, β-interferon, and paclitaxel treated mammary gland tissues showed significantly less staining for Bcl2 expression concluding the possible role of DEAE-Dextran and β-interferon in apoptosis (**Figures 4M,N**).

As a confirmatory analysis, we carried out the flow cytometry analysis since novel approaches have fostered remarkable progress in our understanding of cancer biology, one of which being the recognition that resistance to cell death- particularly apoptotic cell death- is a relevant aspect of both tumorigenic as well as the development of resistance to anticancer drugs (Okada and Mak, 2004). Data analyzed showed that DEAE-Dextran exhibited apoptosis with time at various doses in comparison to β-interferon at the same doses (**Figures 5H–J**). It is hence evident that DEAE-Dextran exerts apoptotic activity which could be the possible mechanism of anti-cancer effect produced till date in the various experimental models. However, the exact pathway of apoptotic death is yet to be analyzed and could be a further keen research area for determining the mechanistic approach of DEAE-Dextran in triple negative breast cancer.

## Conclusion

As per the various investigations carried out in the present study, DEAE-Dextran not only exhibits excellent cytotoxic, anti-oxidant and anti-proliferative activity, but also shows its role as an anti-angiogenic agent. Its β-interferon inducing capacity enables a natural defense mechanism within the body which accounts for the anti-angiogenic action. Also, DEAE-Dextran acts a dual VEGF and NOTCH1 antagonist, which confirms its role in the angiogenesis pathway. Moreover, DEAE-Dextran initiates apoptosis as a means of its anti-tumor activity in MDA-MB-231 cells showing that DEAE-Dextran has multiple pathways for its anti-cancer activity and can therefore be an emerging molecule for the targeted therapy of TNBC. DEAE-Dextran, as a promising molecule could be the future in TNBC management with the in-depth signaling research to follow in the coming era.

## Author Contributions

AB, BV, and SP contributed to the study design and interpretation. AB and BV contributed to the data interpretation and manuscript drafting. SP contributed to the final approval of manuscript. AB, BV, and SP are all accountable for the accuracy and originality of the work in the manuscript.

## Conflict of Interest Statement

The authors declare that the research was conducted in the absence of any commercial or financial relationships that could be construed as a potential conflict of interest.
