# Corrigendum: Role of β-interferon inducer (DEAE-Dextran) in tumorigenesis by VEGF and NOTCH1 inhibition along with apoptosis induction

**DOI:** 10.3389/fphar.2024.1433625

**Published:** 2024-12-13

**Authors:** Anita K. Bakrania, Bhavesh C. Variya, Snehal S. Patel

**Affiliations:** ^1^ Department of Pharmacology, Institute of Pharmacy, Nirma University, Ahmedabad, India; ^2^ Zydus Research Centre, Ahmedabad, India

**Keywords:** DEAE-Dextran, β-interferon, TNBC, anti-proliferative, apoptosis, angiogenesis, VEGF, NOTCH1

In the published article, there was an error in (Figures 2, 4) as published. These errors occurred in preparation of composite figures from individual images, which were inadvertently placed. The corrected (Figures 2, 4) appear below.

**FIGURE 2:**
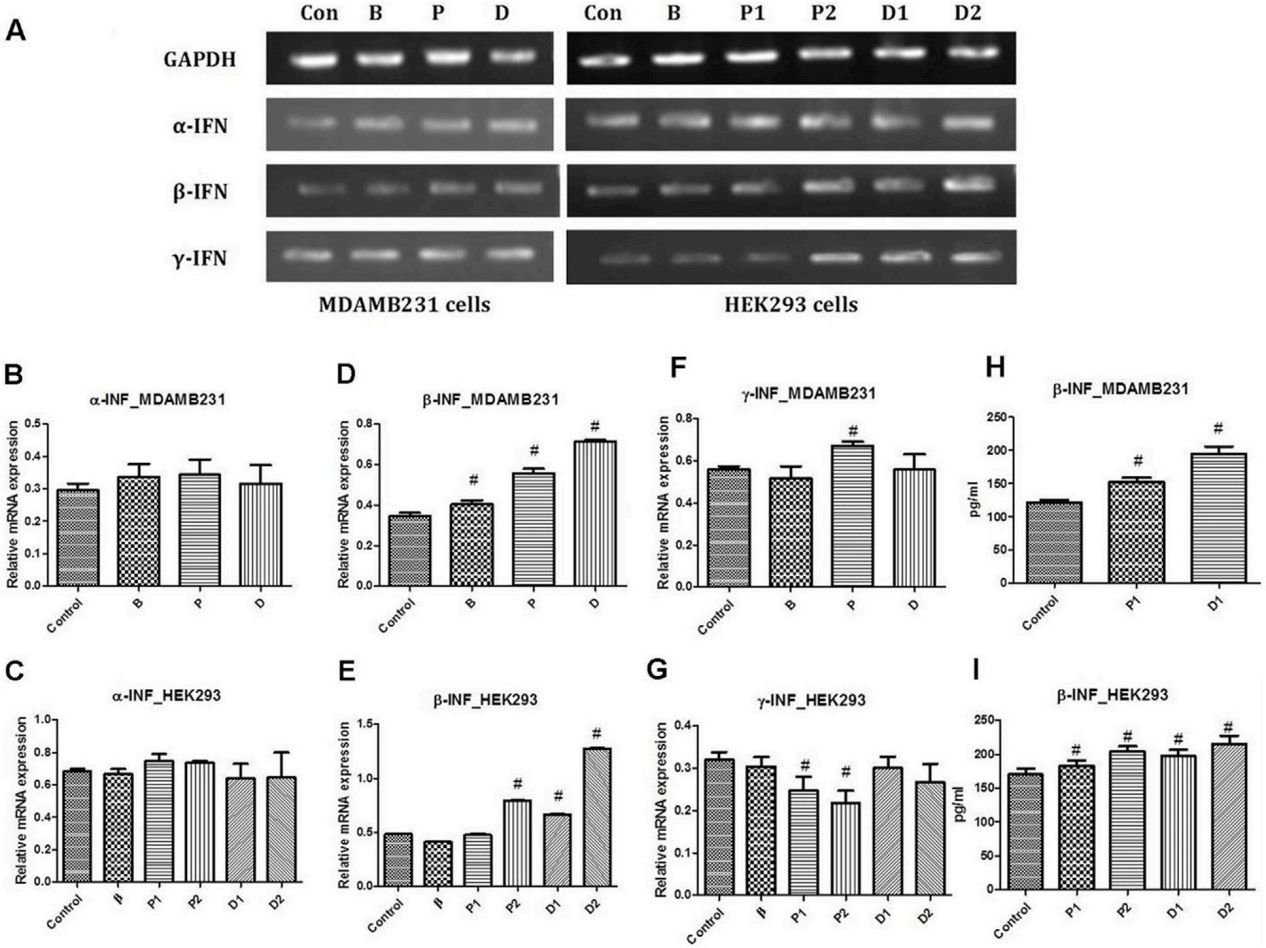
**(A)** Representative PCR bands in various treated cells, **(B)** α-interferon gene expression study in MDA-MB-231 cells, **(C)** α-interferon gene expression study in HEK293 cells, **(D)** β-interferon gene expression study in MDA-MB-231 cells, **(E)** β-interferon gene expression study in HEK293 cells, **(F)** γ-interferon gene expression study in MDA-MB-231 cells and **(G)** γ-interferon gene expression study in HEK293 cells. Determination of β-interferon release in various cell lines; **(H)** β-interferon release in MDA-MB-231 cell line and **(I)** β-interferon release in HEK293 cell line. ^#^ Significantly different from control (*P* < 0.05), Values expressed as Mean ± SEM. Control and con – untreated HEK293 and MDA-MB-231 cells, P1 and P – 1 μM paclitaxel, P2 – 5 μM paclitaxel, D1 and D – 1 μM DEAE-Dextran, D2 – 5 μM DEAE-Dextran, B and β – β-interferon treated cells.

**FIGURE 4:**
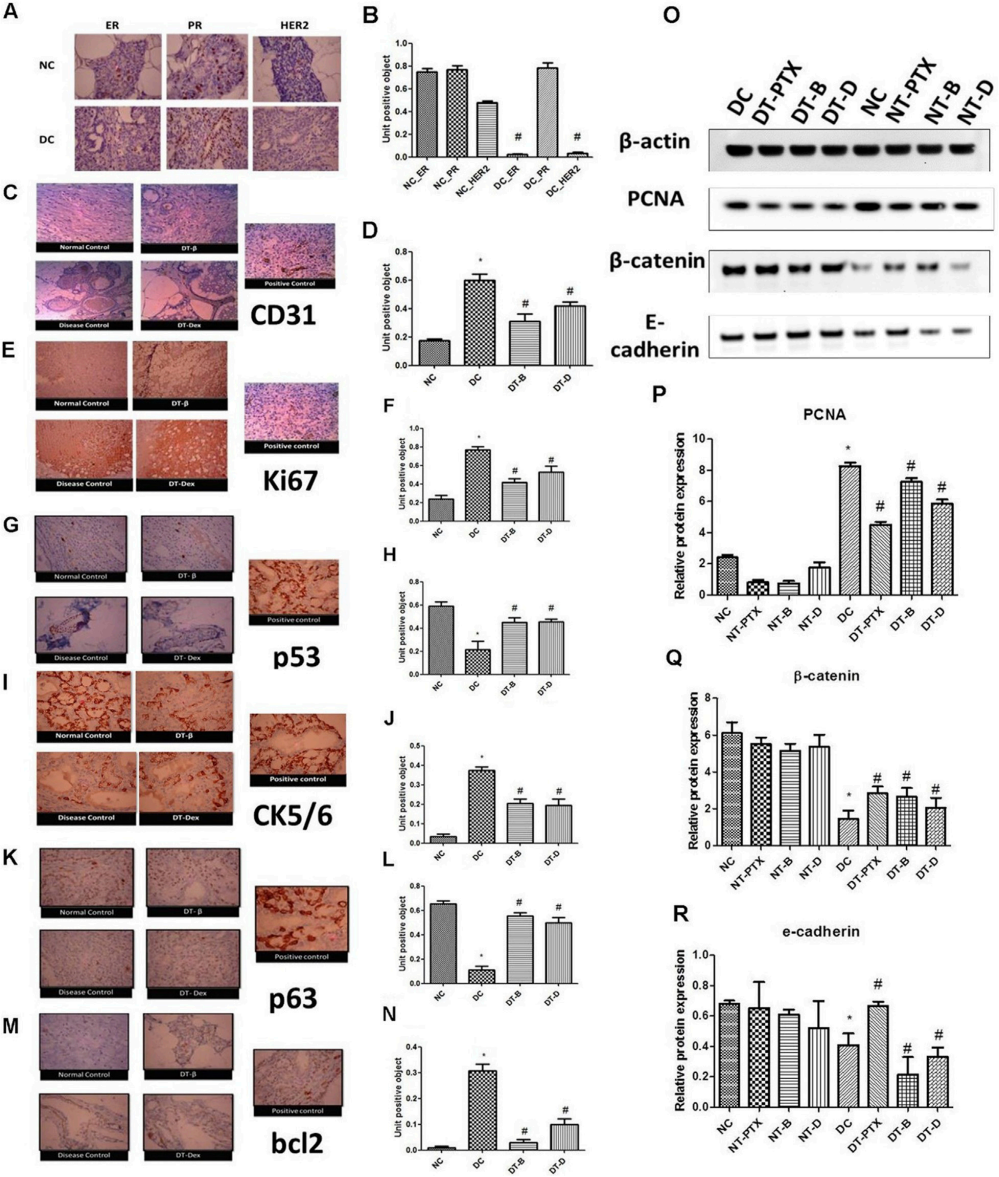
**(A, B)** Immunohistochemistry studies for ER, PR, and HER2 in DMBA induced mammary gland model. NC – control rats, DC – positive control, ER – estrogen antibody staining, PR – progesterone antibody staining, HER2 – HER2 antibody staining, magnification X100. **(C, D)** Immunohistochemistry studies of CD31 in DMBA induced mammary gland model. Positive control of liver section was used, magnification X100. **(E, F)** Immunohistochemistry studies of ki67 in DMBA induced mammary gland model. Positive control of breast carcinoma was used, magnification X100. **(G, H)** Immunohistochemistry studies of p53 in DMBA induced mammary cancer model. Positive control breast cancer sections were used, magnification X100. **(I, J)** Immunohistochemistry studies of CK5/6 in DMBA induced mammary cancer model. Positive control of lung squamous cell carcinoma slide was used, magnification X100. **(K, L)** Immunohistochemistry studies of p63 in DMBA induced mammary cancer model. Positive control as breast cancer section was used, magnification X100. **(M, N)** Immunohistochemistry studies of bcl2 in DMBA induced mammary gland model. Positive control of tonsil section was used. Control animals, positive control, DT-B – rats treated with β-interferon, DT-Dex – rats treated with DEAE-Dextran, magnification X100. Determination of protein expression by Western blot analysis; **(O)** representative Western blot bands, **(P)** determination of PCNA protein expression, **(Q)** determination of β-catenin protein expression, and **(R)** determination of E-cadherin protein expression. ∗Significantly different from control animals (*P* < 0.05), ^#^ Significantly different from positive control (*P* < 0.05), each group consists of six animals, Values expressed as Mean ± SEM. NC – Control animals, DC – positive control, DT-D – 100 mg/kg DEAE-Dextran treated, DT-PTX – 30 mg/kg paclitaxel treated, DT-B – β-interferon treated, NT-D100 – normal treated with 100 mg/kg DEAE-Dextran, NT-PTX – normal treated with 30 mg/kg paclitaxel and NT-B – normal treated with β-interferon.

The authors apologize for this error and state that this does not change the scientific conclusions of the article in any way. The original article has been updated.

